# Oxycodone versus other opioid analgesics after laparoscopic surgery: a meta-analysis

**DOI:** 10.1186/s40001-020-00463-w

**Published:** 2021-01-09

**Authors:** Yan Li, Zhi Dou, Liqiang Yang, Qi Wang, Jiaxiang Ni, Jun Ma

**Affiliations:** 1 Center for Anesthesiology, Beijing Anzhen Hospital, Capital Medical University, No. 2 Anzhen Road, Chaoyang District, Beijing, 100029 China; 2 Department of Anesthesiology, The People’s Hospital of Jizhou District, Tianjin, 301900 Tianjin China; 3 Department of Pain Management, Xuanwu Hospital, Capital Medical University, Beijing, 100053 China

**Keywords:** Meta-analysis, Oxycodone, Laparoscopic surgery, Postoperative visceral pain

## Abstract

**Background:**

Intravenous opioids are administered for the management of visceral pain after laparoscopic surgery. Whether oxycodone has advantages over other opioids in the treatment of visceral pain is not yet clear.

**Methods:**

In this study, the analgesic efficiency and adverse events of oxycodone and other opioids, including alfentanil, sufentanil, fentanyl, and morphine, in treating post-laparoscopic surgery visceral pain were evaluated. This review was conducted according to the methodological standards described in the Cochrane Handbook for Systematic Reviews of Interventions and the Preferred Reporting Items for Systematic Reviews and Meta-analysis statement. The PubMed, Embase, and Cochrane databases were searched in December 2019.

**Results:**

Ten studies were included in this review. The sample size was 695 participants. The results showed that compared with morphine and fentanyl, oxycodone had a more potent analgesic efficacy on the first day after laparoscopic surgery, especially during the first 0.5 h. There was no significant difference in sedation between the two groups. Compared to morphine and fentanyl, oxycodone was more likely to lead to dizziness and drowsiness. Overall, patient satisfaction did not differ significantly between oxycodone and other opioids.

**Conclusions:**

Oxycodone is superior to other analgesics within 24 h after laparoscopic surgery, but its adverse effects should be carefully considered.

## Background

Visceral pain is one of the most frequent reasons that patients seek medical attention after laparoscopic surgery [5]. Opioids are the most commonly provided analgesics for postoperative visceral pain, as they can be used prior to the completion of the operation or in the patient-controlled analgesia (PCA) pump after surgery [1]. However, which kind of opioid is most appropriate is still controversial.

Oxycodone is a semisynthetic drug that is derived from thebaine, an opium alkaloid, and acts as a μ-opioid receptor agonist by affecting the central nervous system. Experiments in rodents suggest that oxycodone also has an effect on the κ-opioid receptor, which is believed to inhibit visceral pain in the visceral nervous system [22]. In animal experiments and clinical observations, oxycodone may occasionally be superior to morphine and fentanyl in the treatment of visceral pain [23]. However, by conducting a meta-analysis, one can detect treatment effects with greater statistical power and estimate these effects with greater precision [24]. To indirectly compare existing evidence on the efficacy of oxycodone and other opioids used in postoperative pain management after laparoscopic surgery, a meta-analysis was performed.

## Methods

### Design

A meta-analysis was performed in this study.

### Data sources

The PubMed, Excerpta Medica (EMBASE), and Cochrane Library databases were searched for trials published from the database inception to December 2019, with no language restrictions. The reference lists of the included studies and relevant reviews were also searched by hand. The search terms included relevant terms and medical subject headings related to oxycodone, laparoscopic surgery, and randomized-controlled trials (RCTs).

The search strategy for each database is presented in Appendix 1.

### Inclusion criteria

The types of studies included were RCTs.

The participants in the studies were patients with a clinical diagnosis of visceral pain after laparoscopic surgery.

The types of interventions were oxycodone versus other opioids including alfentanil, sufentanil, fentanyl, and morphine.

The outcomes included pain intensity measured by the visual analogue scale (VAS) or numeric rating scale (NRS), sedation status, adverse events, and patient satisfaction measured by validated scales. We defined pain intensity as the primary endpoint, and sedation and other adverse events as the secondary endpoints.

### Study screening

All retrieved studies were imported into Endnote X7 (Thomson ResearchSoft, Stanford, CT). To ensure a high level of confidence between researchers, we conducted a pilot test on literature screening. Two researchers independently reviewed the titles and abstracts of the studies and selected studies that met the eligibility criteria. Then, the full texts of all the studies that met the requirements were reviewed.

### Data collected

Using a standardized data sheet in Microsoft Excel 2013 (Microsoft Corp, Redmond, WA, http://www.microsoft.com), two investigators independently extracted data on the study characteristics (e.g., the first author’s name, publication year, region where the study was conducted), characteristics of the study subjects (e.g., number of participants, sex distribution), intervention details (e.g., treatment and comparisons), and outcome variables (e.g., adverse events). Any discrepancies observed in the data extracted by the two investigators were resolved by consensus.

### Risk of bias of individual studies

The risk of bias of the included RCTs was assessed according to the Cochrane Handbook, version 5.1.0 [8], and the aspects assessed included the method of random sequence generation (selection bias), allocation concealment (selection bias), blinding (performance bias and detection bias), incomplete outcome data (detection bias), selective reporting (detection bias), and other bias. We considered the risk of bias to be low, high, or unclear. The risk of bias assessment was completed by two independent reviewers, and conflicts were resolved by a third reviewer.

### Meta-analysis

A meta-analysis was conducted using RevMan 5.3 software. The combined risk ratio (RR) and the 95% confidence interval (CI) were calculated for the dichotomous data. The heterogeneity of the therapeutic effects in the trials was assessed by *χ*^2^ and *I*^2^. If there was no statistical heterogeneity (the *p* value was ≥ 0.1 and *I*^2^ ≤ 50%), the Mantel–Haenszel fixed-effects model was used for the meta-analysis [8]. Otherwise, we explored the potential causes of heterogeneity through subgroup analysis and meta-regression. If no clinical heterogeneity was detected, the meta-analysis was performed using the Mantel–Haenszel random-effects model.

## Results

### Literature selection

**Fig. 1:**
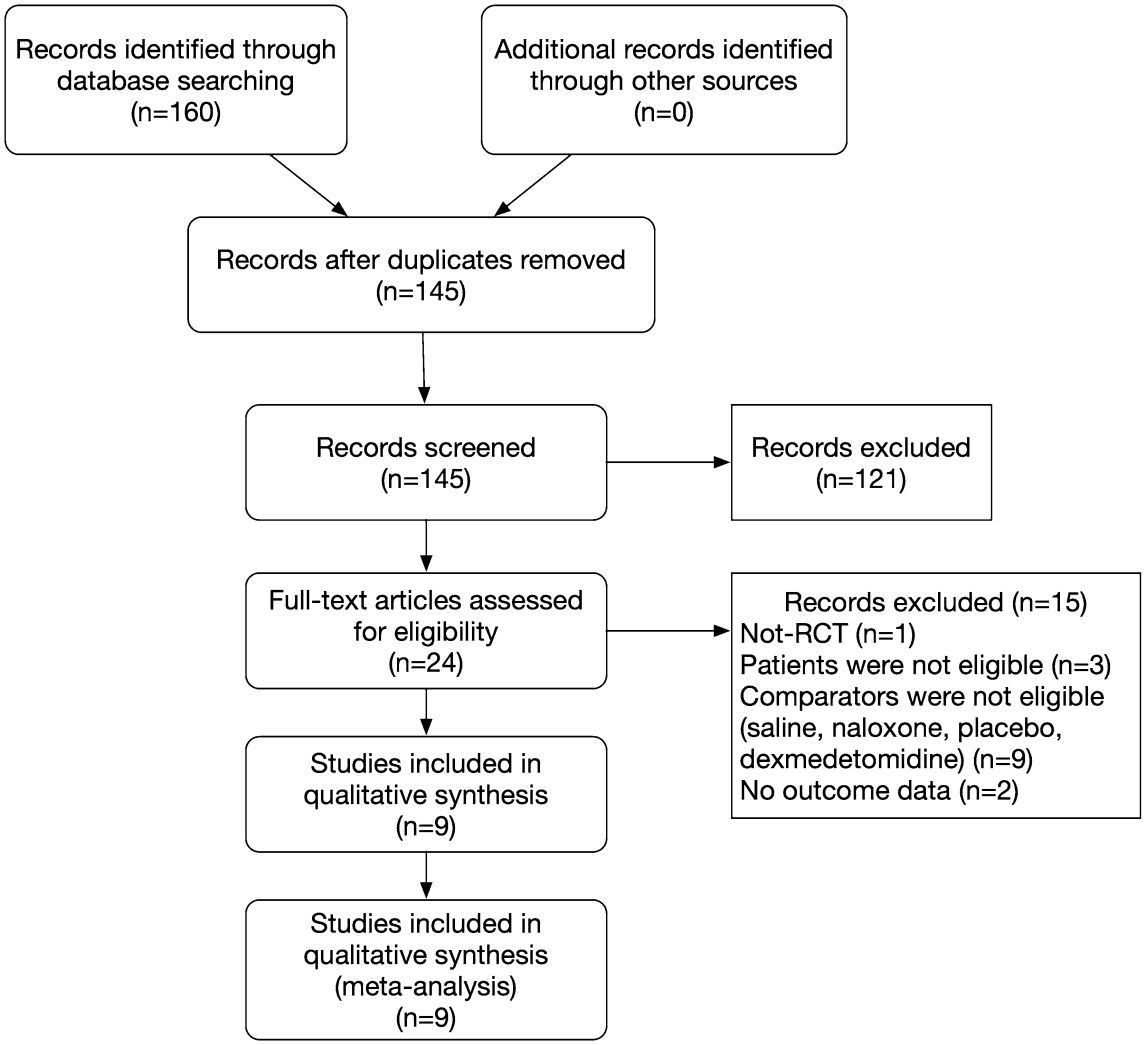
Study screening flow diagram

The search strategy and selection process for the published articles are described in Fig. 1. A total of 145 studies were identified in the search. Of them, 122 articles were excluded after the titles and abstracts were screened. The remaining 23 studies concerning oxycodone for visceral pain after laparoscopic surgery were assessed. Among them, 15 studies were excluded, because they were non-randomized-controlled trials (*n* = 1), the patients did not meet our inclusion criteria (*n* = 3), the comparator did not meet our inclusion criteria (*n* = 9), and the outcomes reported did not meet our inclusion criteria (*n* = 2). A total of nine studies [3, 4, 7, 9, 10, 12–15, 20] met our inclusion criteria.

### Characteristics of the included studies

A total of 695 participants (including 347 oxycodone subjects and 348 controls) met the inclusion criteria and were included in the data analysis; the study design and location, characteristics of the patients (diagnosis, duration of surgery, duration of anaesthesia, American Society of Anesthesiologists (ASA) physical status I/II), and details about the interventions and measured outcomes are presented in Table 1.

### Risk of bias results for the individual studies

**Fig. 2:**
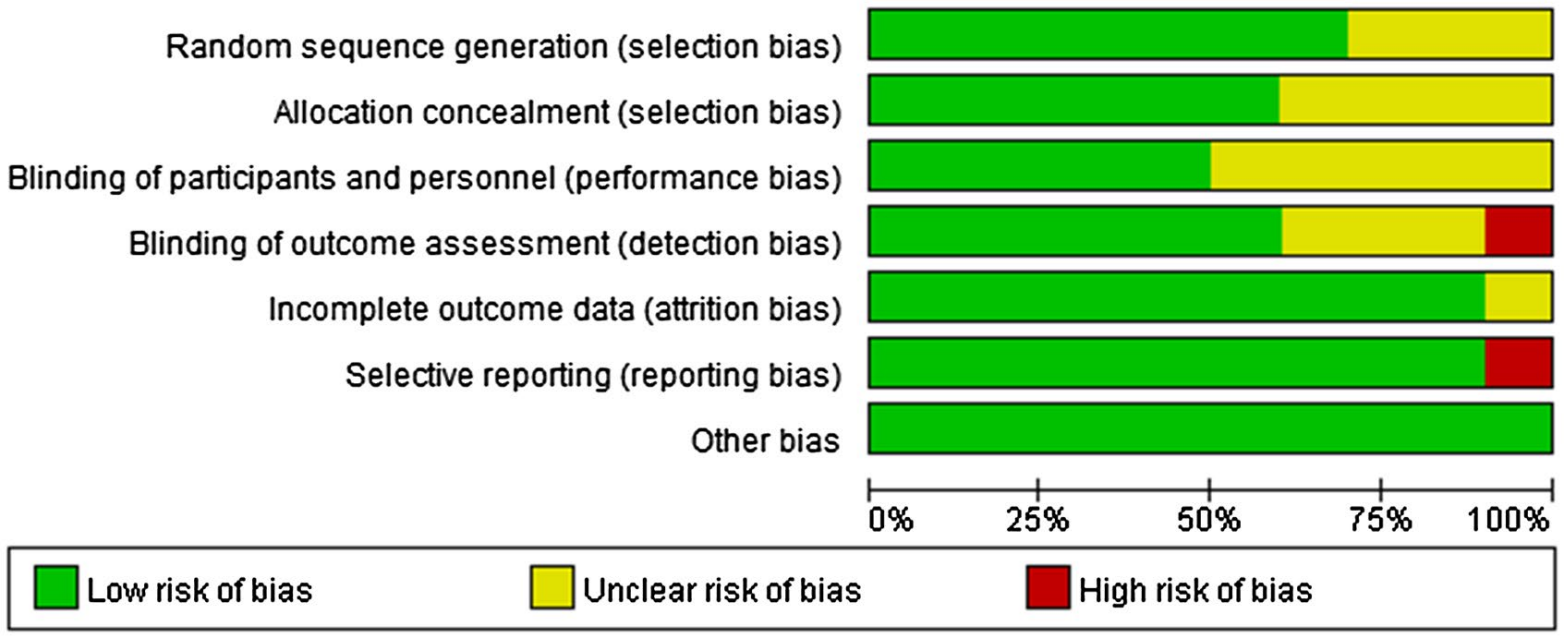
Risk of bias of included studies

The risk of bias results for the included studies determined according to the Cochrane risk of bias tool are provided in Fig. 2. Of the nine studies that were included, six studies were rated as having a low risk of bias regarding randomization, as they used computer-generated random number sequences. Three studies [9, 10, 20] did not describe the method of randomization. Most studies stated that allocation concealment was conducted; however, four studies [9, 10, 14, 20] did not report this information. Fewer than half of the included studies stated that the participants and personnel were blinded. The other five studies [9, 10, 14, 15, 20] did not report this information. For blinding of the outcome assessor, one study [13] stated that some of the outcome assessors knew the group assignments during treatment, so it was rated as having a high risk of bias in this domain. One study [15] did not report data for some measured outcomes, including nausea, vomiting, or itching; therefore, it was rated as having a high risk of selective reporting bias.

### Meta-analysis

#### Pain intensity

**Fig. 3:**
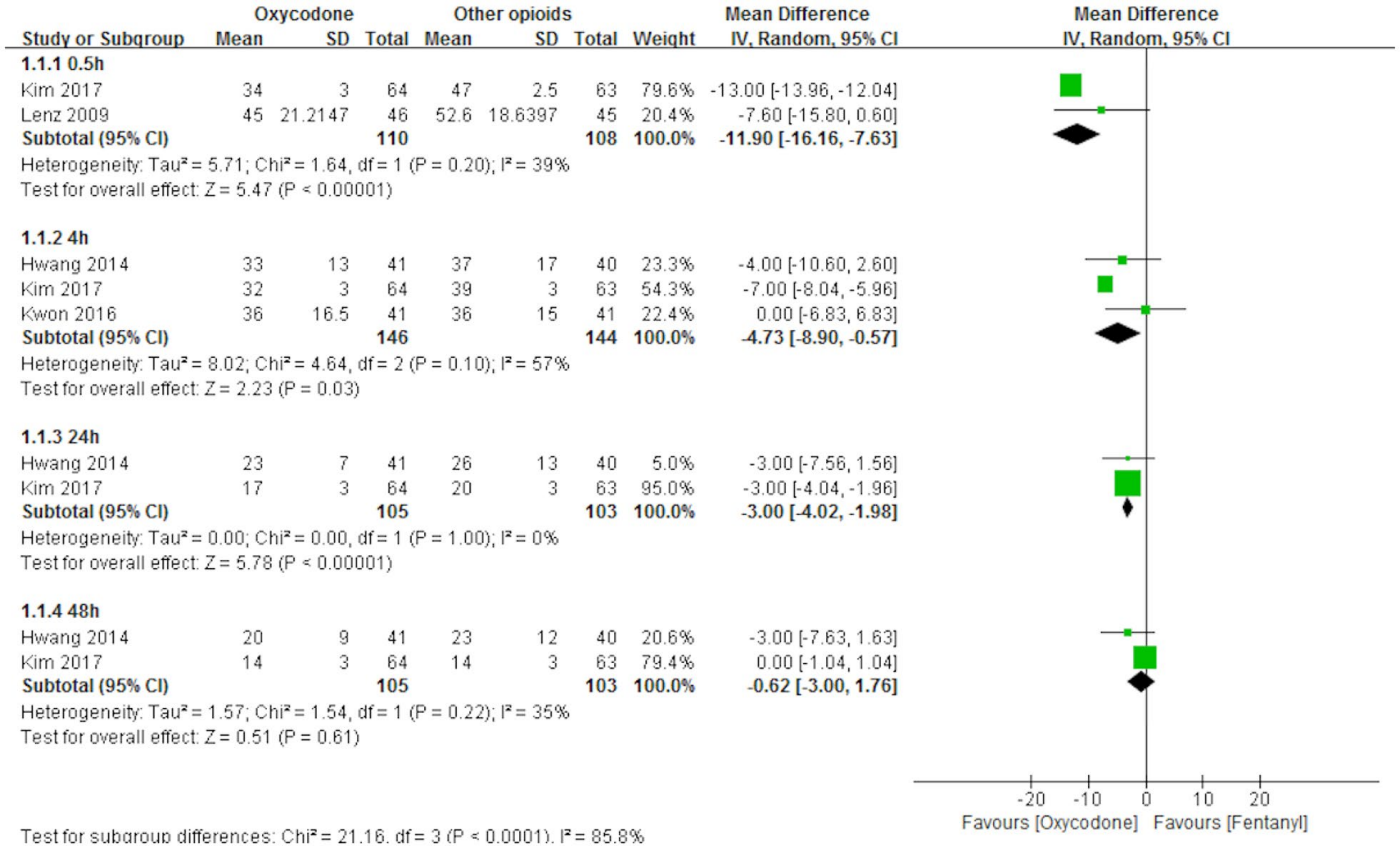
Meta-analysis of pain intensity. *CI* confidence interval

Nine studies [3, 4, 9, 10, 12–15, 20] measured pain intensity by the VAS or NRS. However, data from only four of them [9, 12, 14, 15] were included in the meta-analysis. The results showed that oxycodone significantly reduced pain intensity compared with other opioids (fentanyl, alfentanil, or morphine) at 30 min (2 RCTs, *N* = 218, MD − 11.9, 95% CI -16.16 to − 7.63), 4 h (3 RCTs, *N* = 290, MD − 4.73, 95% CI − 8.9 to − 0.57), and 24 h postoperatively (2 RCTs, *N* = 208, MD − 3.00, 95% CI − 4.02 to − 1.98) but not at 48 h postoperatively (2 RCTs, *N* = 208, MD − 0.62, 95% CI − 3.00 to 1.76) (Fig. 3). The data of the other five studies were not included in the meta-analysis, because the data were skewed. These results are consistent with those reported by Kim et al. [10], Choi et al. [3], and Park et al. [20] which concluded that oxycodone and fentanyl have equal effectiveness in relieving postoperative pain. Choi et al. [4] found that the pain intensity in the oxycodone group was significantly lower than that in the fentanyl group at 0.5 h postoperatively, but this effect did not last longer than 0.5 h. Koch 2008 [13] stated that the intensity of deep abdominal pain was significantly lower in the oxycodone group upon arrival, after 30, 60, and 90 min, and upon discharge from the PACU.

### Sedation

**Fig. 4:**
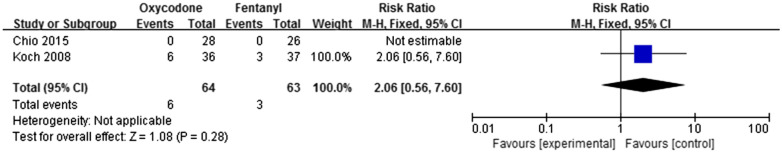
Meta-analysis of sedation score at 2 (occasionally drowsy, easily aroused). *CI* confidence interval

Four studies [4, 9, 13, 15] reported this outcome. Choi et al. [4] and Koch et al. [13] used the following methods to assess sedation: “S, asleep, easily aroused; 1, awake and alert; 2, occasionally drowsy, easily aroused; 3, frequently drowsy, falls asleep during conversation; 4, somnolent, minimal or no response to stimulation”. The meta-analysis showed that there were no differences between oxycodone and fentanyl, as the sedation scores were 2 (2RCTs, *N* = 127, RR 2.06, 95% CI 0.56–7.60, Fig. 4). Both studies reported that no patients had a sedation score of 3 or 4. Two studies used different measurements to evaluate the sedation effects. Hwang et al. [9] also concluded that the sedation level was similar between the oxycodone and fentanyl groups. However, Lenz et al. [15] found a different result: the sedation level was significantly lower in the oxycodone group than in the morphine group (*P* = 0.006).

### Adverse events

**Fig. 5:**
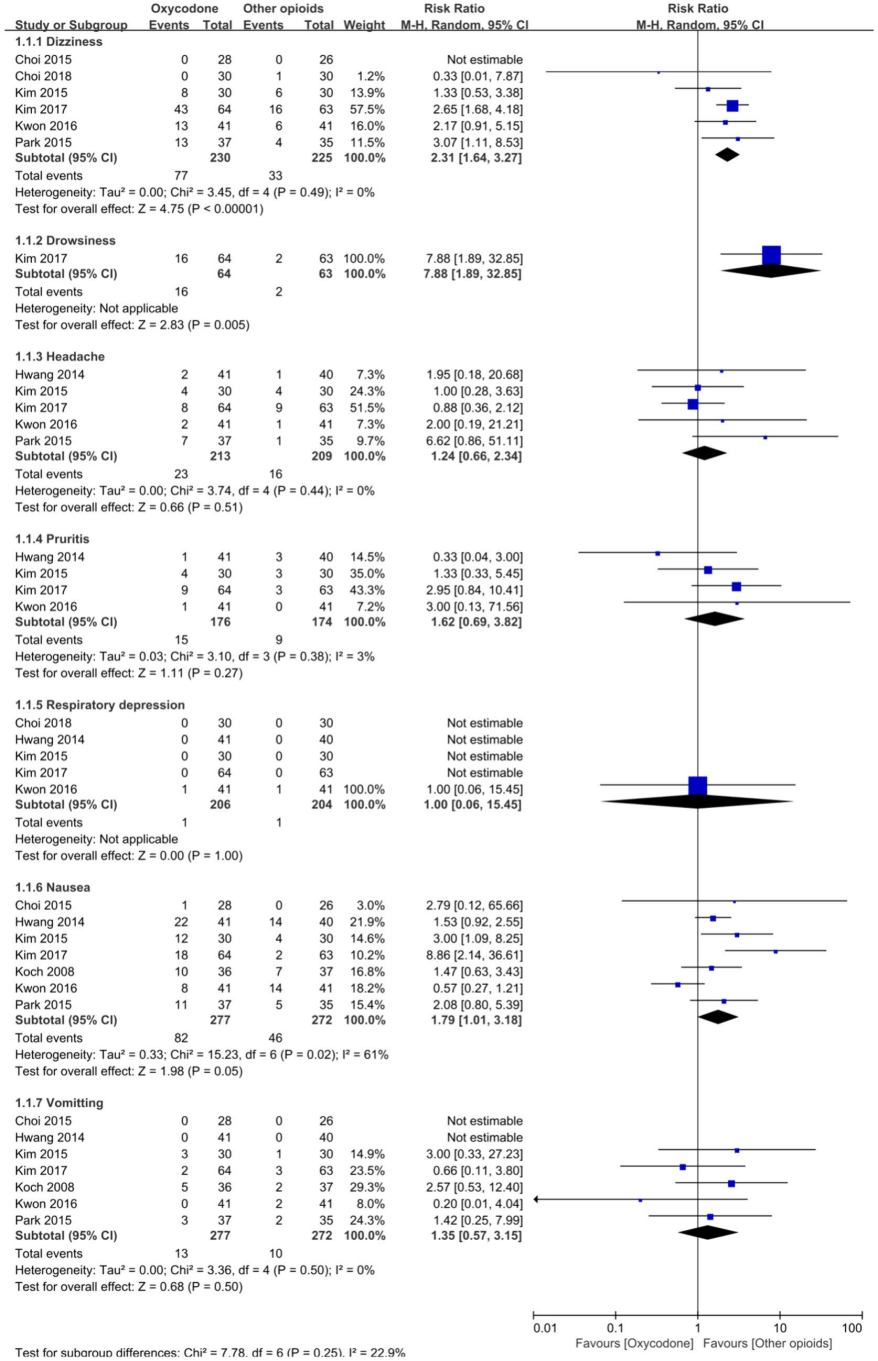
Meta-analysis of adverse events. *CI* confidence interval

All studies reported adverse events. Oxycodone may induce a higher risk of dizziness (6 RCTs, *N* = 455, RR 2.31, 95% CI 1.64–3.27), drowsiness (1 RCT, *N* = 127, RR 7.88, 95% CI 1.89–32.85), and nausea (7 RCT, *N* = 549, RR 1.79, 95% CI 1.01–3.18). There were no differences between groups in the risk of headache, pruritus, respiratory depression, or vomiting (Fig. 5).

### Patient satisfaction

**Fig. 6:**
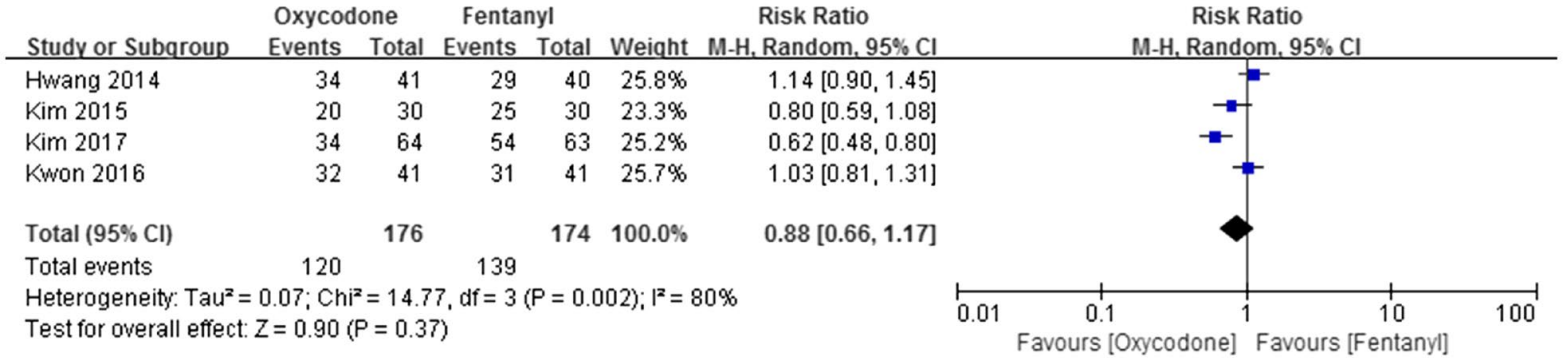
Meta-analysis of patient satisfaction. *CI* confidence interval

Patient satisfaction was classified into four levels: very satisfied, satisfied, neutral, and dissatisfied. A meta-analysis was performed to assess the number of patients who were satisfied or very satisfied in the two groups. The results from four studies [9, 10, 12, 14] showed that there were no significant differences between oxycodone and other opioids in this outcome (4 RCTs, *N* = 350, RR 0.88, 95% CI 0.66–1.17, Fig. 6**).**

## Discussion

### Summary of findings

A total of 9 studies, including 695 patients, were included in this meta-analysis to compare the analgesic effect of oxycodone and other opioids, including fentanyl, morphine, sufentanil, and alfentanil. Most of the included studies reported the efficacy of oxycodone and indicated that it was superior to other analgesics in treating visceral pain within 24 h after laparoscopic surgery [9, 10, 12, 14, 15]. However, there was no significant difference in the pain scores between the oxycodone group and other opioid groups at 48 h after surgery. There does not appear to be a clear consensus regarding the findings on the sedation level, adverse events, or patient satisfaction. However, this finding suggests that oxycodone may induce a higher risk of dizziness and drowsiness than do other opioids. We also found no significant differences in patient satisfaction between these other opioids and oxycodone.

### Quality of the evidence

The quality of the evidence was fair. Most studies were rated as having a low risk of bias regarding randomization, allocation concealment, blinding, the attrition rate, and selective reporting. Only two studies (Koch, Lenz) were rated as having poor quality, owing to issues of imprecision (small sample size and a sparse number of events observed) and risk of bias (unclear reporting of allocation concealment and blinding).

### Analgesic efficacy

Postoperative pain after laparoscopic surgery consists of three components: incisional pain (somatic), deep abdominal pain (visceral), and inflammatory pain after carbon dioxide is absorbed by the peritoneum (also referred to as visceral pain) [13]. This study used the ideal clinical design to test the effectiveness of visceral pain treatments, and the somatic pain component was minimized.

Four of the included studies showed that oxycodone is more potent in the treatment of visceral pain than is morphine or fentanyl during the first 0.5 h after surgery. In these studies, the intensity of analgesic drugs peaked at this time point. Fentanyl has a rapid onset of action (5–7 min), which is much faster than that of oxycodone (10–15 min) [11]. Although morphine is considered to be a slower acting drug, it was given to patients 10–15 min before the end of surgery in Lens’s study [15]. Therefore, the onset time cannot be used to explain the difference in their initial pain relief.

The analgesic effects of oxycodone may be explained by a specific mechanism. Several recent studies have suggested that oxycodone attenuates visceral pain better than do other opioids [16, 18, 23]. Oxycodone has a proposed effect on the κ-opioid receptor, which reflects a different pharmacological profile from those of other opioids. κ-opioid receptors on peripheral nerves in the gut have been suggested as important components in anti-nociception in the visceral pain system [25]. The analgesic effect of oxycodone correlates with the plasma concentration, indicating an effect in the periphery that is perhaps mediated via κ-receptors [26].

This meta-analysis also showed that at 4 h and 24 h, the analgesic effect of oxycodone is superior to that of other opioids, regardless of whether a single dose was administered at the end of the surgery or the dose was administered using a PCA pump. These findings indicate that oxycodone is more potent than are other opioids in the treatment of postoperative visceral pain with the equivalent dose. However, the analgesic advantage of oxycodone did not last for more than 48 h after surgery, regardless of whether a single dose [9] or a dose via a PCA pump [12] was administered. A possible explanation is that the pain intensity 48 h after such a minor surgery may be too low to yield a significant difference in pain scores [6]. Moreover, it should be noted that because the included studies generally have low pain level, when oxycodone is used for postoperative analgesia in other higher level pain surgeries, its analgetic effect on visceral pain is not yet known, and further research is needed.

### Safety evaluation

Sedation is an important indicator for evaluating the safety of a drug for postoperative analgesia [17]. Lenz et al. found that the sedation level was significantly lower in the oxycodone group than in the morphine group [15]. The meta-analysis showed that the oxycodone groups had similar sedation levels to the morphine and fentanyl groups, and there was no incidence of excessive sedation or respiratory depression in any of the groups.

According to previous studies, the adverse effects associated with opioid use include constipation, nausea, vomiting, drowsiness, dizziness, and pruritus [2, 19]. The specific incidence of the adverse effects varies greatly, depending on the dosage. A higher incidence of dizziness and nausea was reported with oxycodone than with fentanyl and morphine in our study. The precise mechanism of opioid-induced dizziness is unknown. Vestibular sensitivity caused by opioids activating μ receptors in the vestibular epithelium may be involved [11]. Nevertheless, the potential causes of the varied incidence of dizziness still need to be explored further. Among previous studies, the reported incidence of side effects differed widely, probably because most studies were designed to have a statistical power sufficient for investigating analgesic efficacy rather than differences in side effects. In addition, since laparoscopic abdominal surgery often affects intestinal function, it is also important to understand the difference between intestinal paralysis and constipation. However, the included studies did not report these two results.

### Strength and limitations

This meta-analysis has several strengths. First, our search strategy was developed by an information specialist to avoid missing any relevant trials. Second, two reviewers screened and extracted the data to reduce system error in the fabrication process. Similar to other studies, our meta-analysis also had some limitations: there were only a small number of clinical trials available, which contributed a relatively small sample size for the meta-analysis. Second, age and gender may also alter opioid pharmacokinetics and influence pain. The mean age was relatively high in the present patient population (40–69) and the number of female patients accounted for a large proportion in three included studies [3, 13, 14]. Third, two studies had excluded patients undergoing chronic pain medications [9, 14], but for other studies, if patients have chronic pain disease before surgery, or have long-term regular use of analgesics, anticonvulsants, and antidepressants, it may have an impact on the results of the study. Without a washout period, these drugs may have a synergistic effect with opioids, resulting in lower pain scores. Fourth, publication bias may have resulted in the overestimation of some outcomes, as positive results are more likely to be published than are negative ones [21].

## Conclusion

Choosing the best opioid for postoperative visceral pain treatment is complicated, as no universally accepted ‘‘gold standard’’ exists. The results of this meta-analysis suggest that oxycodone is superior to other analgesics within 24 h after laparoscopic surgery. However, in some cases, even when it is effective, its incidence of adverse reactions, especially dizziness, is high. Clinicians must choose appropriate opioids based on their clinical judgement and adjust the dose as needed. To obtain the best clinical evidence, it is necessary to perform more in-depth research in this field.

## Data Availability

The datasets used and analysed for the current study are available from the corresponding author upon reasonable request.

## Appendix 1

EMBASE.COM

PubMed

The Cochrane Library

## Abbreviations

PCA: Patient-controlled analgesia
RCTs: Randomized-controlled trials
VAS: Visual analogue scale
NRS: Numeric rating scale
RR: Risk ratio
CI: Confidence interval
ASA: American Society of Anesthesiologists
