# Correction to: Oxycodone versus other opioid analgesics after laparoscopic surgery: a meta-analysis

**DOI:** 10.1186/s40001-021-00486-x

**Published:** 2021-02-05

**Authors:** Yan Li, Zhi Dou, Liqiang Yang, Qi Wang, Jiaxiang Ni, Jun Ma

**Affiliations:** 1 Department of Anesthesiology, The People’s Hospital of Jizhou District, Tianjin, 301900 Tianjin China; 2 Center for Anesthesiology, Beijing Anzhen Hospital, Capital Medical University, No. 2 Anzhen Road, Chaoyang District, Beijing, 100029 China; 3 Department of Pain Management, Xuanwu Hospital, Capital Medical University, Beijing, 100053 China

## Correction to: Eur J Med Res (2021) 26:4 https://doi.org/10.1186/s40001-020-00463-w

After online publication of the article [1], the first author would like to change the order of his affiliations. The corrected order of affiliations is given in this Correction article. The original article has been corrected.
